# Supplementary material for: Corneal endothelial cell density loss following glaucoma surgery alone or in combination with cataract surgery: a systematic review protocol
Source: BMJ Open. 2021 Sep 13;11(9):e050992. doi: 10.1136/bmjopen-2021-050992 (PMC8438923; doi:10.1136/bmjopen-2021-050992)
Supplement: Supplementary data [file bmjopen-2021-050992supp001.pdf]

Appendix I: Search strategy

EMBASE

Search conducted in May 2020

Search	Query	Records Retrieved
1	exp glaucoma/	77252
2	glaucoma*.mp.	84904
3	1 or 2	88723
4	exp eye surgery/ or exp surgery/	4584292
5	(Surg* or procedure* or operat* or incision* or invasive* or penetrat* or Drainage implant surg* or Electrocautery or Cyclophotocoagulation*).mp.	6437635
6	4 or 5	7735880
7	endothelial* cell*.ab. or endothelial* cell*.ti.	214675
8	3 and 6 and 7	870
9	exp cataract/	54959
10	exp phacoemulsification/	14524

11	(cataract* or pha?oemulsif*).mp. [mp=title, abstract, heading word, drug trade name, original title, device manufacturer, drug manufacturer, device trade name, keyword, floating subheading word, candidate term word]	91182
12	9 or 10 or 11	92259
13	exp eye surgery/ or exp surgery/	4584292
14	(Surg* or procedure* or operat* or incision*).mp.	6019212
15	13 or 14	7359674
16	(micro or small or minimally or minimal).mp.	2452911
17	(microincision* or micro-incision* or incision*).ti.	18429
18	16 or 17	2465927
19	7 and 12 and 15 and 18	315
20	8 or 19	1138
21	limit 20 to (human and english language and yr="2000 -Current")	724

MEDLINE

Search conducted in May 2020

**Search**	**Query**	**Records Retrieved**

1	exp Glaucoma, Angle-Closure/ or exp Low Tension Glaucoma/ or exp Glaucoma/ or exp Glaucoma, Open-Angle/ or exp Glaucoma, Neovascular/ or exp Glaucoma Drainage Implants/	52351
2	glaucoma*.mp.	70177
3	1 or 2	70398
4	exp General Surgery/	38657
5	(Surg* or procedure* or operat* or incision* or invasive* or penetrat* or Drainage implant surg* or Electrocautery or Cyclophotocoagulation*).mp.	4681137
6	4 or 5	4681137
7	exp Endothelial Cells/	58726
8	endothelial* cell*.ab. or endothelial* cell*.ti.	164910
9	7 or 8	178945
10	3 and 6 and 9	509
11	exp Cataract/	28781
12	exp Phacoemulsification/	9762
13	(cataract* or pha?oemulsif*).mp.	72591
14	11 or 12 or 13	72661

15	exp General Surgery/	38657
16	(Surg* or procedure* or operat* or incision*).mp.	4326472
17	15 or 16	4326472
18	(micro or small or minimally or minimal).mp.	1918877
19	(incision* or (microincision* or micro-incision*)).ti.	15328
20	18 or 19	1930443
21	9 and 14 and 17 and 20	218
22	10 or 21	708
23	limit 22 to (english language and humans and yr="2000 -Current")	408

Appendix II: Draft data extraction form

Reviewer name:	
**Citation details:**<br>Authors:<br>Title:<br>Journal:<br>Year:	
**Study details:**<br>Study design:<br>Sample size:<br>Country:	
**Participant characteristics:**<br>Age:<br>Sex:	

**Inclusion criteria**<br>**Exclusion criteria**	
**Surgery type:**<br>**Intervention:**<br>**Comparator(s):**	
**Outcome**: (at all time assessment points)<br>Mean ECD pre-operation:<br>Mean ECD post-operation:<br>Percentage ECD loss:<br>Corneal complications/adverse events:	
