# Supplementary material for: Corneal endothelial cell density loss following glaucoma surgery alone or in combination with cataract surgery: a systematic review protocol
Source: BMJ Open. 2021 Sep 13;11(9):e050992. doi: 10.1136/bmjopen-2021-050992 (PMC8438923; doi:10.1136/bmjopen-2021-050992)
Supplement: Author's manuscript [file bmjopen-2021-050992.draft_revisions.pdf]

BMJ Open is committed to open peer review. As part of this commitment we make the peer review history of every article we publish publicly available.

When an article is published we post the peer reviewers' comments and the authors' responses online. We also post the versions of the paper that were used during peer review. These are the versions that the peer review comments apply to.

The versions of the paper that follow are the versions that were submitted during the peer review process. They are not the versions of record or the final published versions. They should not be cited or distributed as the published version of this manuscript.

BMJ Open is an open access journal and the full, final, typeset and author-corrected version of record of the manuscript is available on our site with no access controls, subscription charges or pay-per-view fees (http://bmjopen.bmj.com).

If you have any questions on BMJ Open's open peer review process please email info.bmjopen@bmj.com

BMJ Open

Corneal endothelial cell density loss following glaucoma surgery alone or in combination with cataract surgery: A systematic review protocol

Journal:	*BMJ Open*
Manuscript ID	bmjopen-2021-050992
Article Type:	Protocol
Date Submitted by the Author:	08-Mar-2021

Complete List of Authors:	Fang, Clarissa E H; University College London Institute of Ophthalmology<br>Khaw, Peng; University College London Institute of Ophthalmology; NIHR Moorfields Biomedical Research Centre<br>Mathew, Rashmi G; University College London Institute of Ophthalmology; Moorfields Eye Hospital NHS Foundation Trust<br>Henein, Christin; University College London Institute of Ophthalmology; NIHR Moorfields Biomedical Research Centre

Keywords:	Glaucoma < OPHTHALMOLOGY, Cataract and refractive surgery < OPHTHALMOLOGY, Ophthalmology < SURGERY

SCHOLARONE™
Manuscripts

I, the Submitting Author has the right to grant and does grant on behalf of all authors of the Work (as defined in the below author licence), an exclusive licence and/or a non-exclusive licence for contributions from authors who are: i) UK Crown employees; ii) where BMJ has agreed a CC-BY licence shall apply, and/or iii) in accordance with the terms applicable for US Federal Government officers or employees acting as part of their official duties; on a worldwide, perpetual, irrevocable, royalty-free basis to BMJ Publishing Group Ltd ("BMJ") its licensees and where the relevant Journal is co-owned by BMJ to the co-owners of the Journal, to publish the Work in this journal and any other BMJ products and to exploit all rights, as set out in our licence.

The Submitting Author accepts and understands that any supply made under these terms is made by BMJ to the Submitting Author unless you are acting as an employee on behalf of your employer or a postgraduate student of an affiliated institution which is paying any applicable article publishing charge ("APC") for Open Access articles. Where the Submitting Author wishes to make the Work available on an Open Access basis (and intends to pay the relevant APC), the terms of reuse of such Open Access shall be governed by a Creative Commons licence – details of these licences and which Creative Commons licence will apply to this Work are set out in our licence referred to above.

Other than as permitted in any relevant BMJ Author's Self Archiving Policies, I confirm this Work has not been accepted for publication elsewhere, is not being considered for publication elsewhere and does not duplicate material already published. I confirm all authors consent to publication of this Work and authorise the granting of this licence.

For peer review only

Title

Corneal endothelial cell density loss following glaucoma surgery alone or in combination with cataract surgery: A systematic review protocol

Authors

Clarissa E H Fang[1], Peng T Khaw[1,2], Rashmi G Mathew[1,3], Christin Henein[1,2]

Affiliations

[1]UCL, Institute of Ophthalmology, London, UK
[2] NIHR Moorfields Biomedical Research Centre, London, UK
[3] Moorfields Eye Hospital NHS Foundation Trust, London, UK

Corresponding author

Christin Henein
c.henein@ucl.ac.uk

I, the Submitting Author has the right to grant and does grant on behalf of all authors of the Work (as defined in the below author licence), an exclusive licence and/or a non-exclusive licence for contributions from authors who are: i) UK Crown employees; ii) where BMJ has agreed a CC-BY licence shall apply, and/or iii) in accordance with the terms applicable for US Federal Government officers or employees acting as part of their official duties; on a worldwide, perpetual, irrevocable, royalty-free basis to BMJ Publishing Group Ltd ("BMJ") its licensees and where the relevant Journal is co-owned by BMJ to the co-owners of the Journal, to publish the Work in BMJ Open and any other BMJ products and to exploit all rights, as set out in our licence.

Authors contribution

CH conceived the idea for the review. CEHF and CH drafted and revised the protocol with suggestions from PTK and RGM who reviewed the protocol and provided feedback on the draft. CEHF constructed the search.

Conflicts of interest

The authors declare no conflict of interest.

Data statement

For peer review only

Supplementary materials, appendix, and dataset will be made available in a data repository.

Patient and Public Involvement

Patients and public were not involved in the development of this protocol.However the James Lind Alliance has prioritised the need to identify safe and effective treatments for glaucoma.

Acknowledgments

The authors would like to acknowledge the funding as mentioned below.

Funding

The authors receive funding from the National Institute for Health Research Biomedical Research Centre at Moorfields Eye Hospital NHS Foundation Trust and UCL Institute of Ophthalmology, the UK Medical Research Council, Moorfields Eye Charity, the Michael and Ilse Katz Foundation, the Helen Hamlyn Trust and Fight for Sight (UK).

Email addresses:

Clarissa E H Fang fangclarissa@gmail.com

Peng T Khaw p.khaw@ucl.ac.uk

Rashmi G Mathew r.mathew@ucl.ac.uk

Christin Henein c.henein@ucl.ac.uk

For peer review only

Ethics and Dissemination

Ethics approval is not required for this review, as it will only include published data. Findings will be published in a peer-reviewed journal and disseminated across ophthalmic networks. We anticipate that the findings of this work will be of interest to multiple stakeholders: people who have undergone glaucoma surgery, eye health professionals, ophthalmic surgeons, device manufacturers and policy makers. It will also inform researchers to where there are gaps in evidence and identify areas for future research.

Abstract

Objective: We aim to systematically assess and compare corneal endothelial cell density (ECD) loss in patients with glaucoma following glaucoma surgery and cataract surgery.

Introduction: Corneal ECD loss may occur due to intraoperative surgical trauma in glaucoma surgery or postoperatively with chronic endothelial cell trauma or irritation. Corneal edema and decompensation after aqueous shunt glaucoma surgery has been reported but the long term ECD loss is still unknown.

Inclusion criteria: Trabeculectomy, glaucoma filtration surgery or microinvasive glaucoma surgery in adults with ocular hypertension, primary and secondary open angle glaucoma, normal tension glaucoma and angle-closure glaucoma. Participants with pre-existing corneal disease will be excluded. Glaucoma laser treatments and peripheral iridotomy will be excluded. The outcomes include preoperative and postoperative corneal ECD, percentage change of corneal ECD and adverse events.

Methods: We will conduct an electronic database search for randomized controlled trials, prospective non-randomized studies, observational studies in MEDLINE, EMBASE, Cochrane Central Register of Controlled Trials (CENTRAL), ClinicalTrials.gov and The International Prospective Register of Systematic Reviews (PROSPERO). Eligibility criteria will include quantitative articles published after and including the year 2000, written in English and containing data on ECD loss. Two independent reviewers will screen titles and abstracts and extract data from full texts, reporting outcomes according to Preferred Reporting Items for Systematic Reviews and Meta-Analyses (PRISMA) guidelines. Data extraction of key characteristics will be completed using customized forms. Methodological quality will be assessed using the Joanna Briggs Institute critical appraisal forms.

Systematic review registration number: PROSPERO CRD42020192303

Keywords: glaucoma surgery, cataract surgery, endothelial cell density, minimally invasive glaucoma surgery, trabeculectomy

Article Summary

For peer review only

Strengths and Limitations

- This protocol adheres to the Preferred Reporting Items for Systematic Reviews and Meta-Analysis protocols and has been published prospectively in PROSPERO database
- The review will comprehensively assess published peer-reviewed manuscripts assessing endothelial cell loss and adverse events following glaucoma surgery
- A potential limitation might be the paucity of high-quality glaucoma surgery trials measuring endothelial cell density loss
- Due to the expected heterogeneity in study methods, it is unlikely that a network meta-analysis will be possible

Word count: 2112 words

For peer review only

Introduction

The corneal endothelium is a monolayer of hexagonal cells lining the posterior corneal surface that regulates stromal hydration. This squamous cell layer does not regenerate and if damaged, the cornea becomes edematous, causing loss of corneal clarity thus compromising vision, negatively affecting quality of life. Physiologically endothelial cell density (ECD) reduces with age at a rate 0.5% per year.[1] Glaucoma is a known condition that predisposes to endothelial cell damage, although the underlying mechanisms are not well elucidated.[2] Certain types of glaucoma are more predisposed to ECD loss such as when there is a sharp rise in intraocular pressure (IOP) in acute angle closure.[3] Glaucoma has also been associated with poor cornea graft survival.[4,5] High levels of IOP as well as long duration of high IOP are associated with more ECD loss.[2,6,7]

Glaucoma surgery is effective in reducing IOP and preventing the progression of optic nerve damage and visual field loss. Trabeculectomy, or bleb filtration surgery, is considered the gold standard and is frequently performed for medically uncontrolled glaucoma. Aqueous drainage shunts have conventionally been used in cases of uncontrolled glaucoma following unsuccessful trabeculectomy. However, some corneal endothelial cell damage after surgery is inevitable. In the literature, corneal edema after aqueous shunt surgery due to corneal decompensation has been reported.[8] Large randomized controlled trials on aqueous shunt surgery; The Ahmed vs Baerveldt Study and Ahmed Baerveldt Comparison Study had an average incidence rate of 12% persistent corneal edema 5 years post-operation, but did not measure or report ECD loss.[9,10] The safety of glaucoma filtration surgery has progressed with the advancement from full-thickness procedures to ab interno surgery and more recently, microinvasive glaucoma surgery (MIGS). MIGS is frequently combined with cataract surgery which in itself is known to cause ECD loss.[11,12] Implantable devices used in glaucoma surgery are manufactured from different materials with varying biocompatibility profiles. The proximity of implants from the endothelial cells, tube stability and micromovements can impact ECD. Recently, there has been a growing use of new ophthalmic implants for treatment of glaucoma. ECD loss is an important

For peer review only - http://bmjopen.bmj.com/site/about/guidelines.xhtml

surrogate marker for device safety and surgical failure. It is used as a requirement for some Food and Drug Administration (FDA) approved investigational device exemption (IDE) trials and an endpoint for post-market approval surveillance studies. In August 2018, the MIGS device Cypass Micro-Stent (Alcon, USA) was voluntarily withdrawn from the market due to safety concerns when it was reported that 27.2% of patients had ECD loss of >30% at 5 years.[13,14] Interestingly, the device was not considered to have continuing ECD loss at 2 years in a previous study.[15]

The common cataract surgery method of phacoemulsification is where ultrasound is used to break down and aspirate the cataractous lens in the eye of a patient, followed by insertion of an intraocular lens. Cataract surgery has evolved in the last decade with new microincisional techniques which aim to cause less damage to the cornea.[16,17] This pertains to glaucoma patients requiring cataract surgery as well.[18] Patients with glaucoma often have co-existing cataracts and will eventually require surgery for one or the other. It is possible to undergo glaucoma surgery and cataract surgery in the same operation. MIGS is frequently combined with cataract surgery. In fact, in the United states, some stents used in Schlemm's canal are only licensed for combination surgery and not for stand-alone procedures. This is reflected in trials where the intervention is combination surgery compared to phacoemulsification alone.[14,15] Combination surgery carries a distinctive risk-benefit evaluation compared to glaucoma surgery alone. Theoretically, further cost and risks of adding a short procedure to an existing operation may be less than those acquired by a separate hospital admission and anesthetic. These combination surgeries may consequently promote a lower threshold for the use of MIGS in clinical practice. However, combination surgery can negatively affect surgical outcome in complicated cases. Since cataract surgery also causes ECD loss, it is an important confounder in many glaucoma surgery trials.

This review focuses on ECD loss from trabeculectomy, glaucoma filtration surgery and MIGS, alone or in combination with cataract surgery. A preliminary search of PROSPERO, MEDLINE, the Cochrane Database of Systematic Reviews and the *JBI Database of Systematic Reviews and Implementation Reports* was conducted and no current or underway systematic reviews on the topic were identified. There is a need to quantitatively and critically appraise available evidence, and to evaluate where gaps exist.

Review question

To determine the extent of endothelial cell density loss following different types of glaucoma surgery alone or in combination with cataract surgery in adults with ocular hypertension, primary and secondary open angle glaucoma, normal tension glaucoma and angle-closure glaucoma.

Inclusion criteria

Participants

For peer review only - http://bmjopen.bmj.com/site/about/guidelines.xhtml

This review will consider studies that include participants with ocular hypertension, primary and secondary open angle glaucoma, normal tension glaucoma and angle-closure glaucoma. There are no restrictions on geographic location, setting or demographic factors. Participants with pre-existing corneal disease such as Fuch's endothelial dystrophy, iridocorneal endothelial syndrome and corneal graft transplant and the pediatric population will be excluded.

Intervention(s)

We will include incisional glaucoma surgery such as trabeculectomy, glaucoma drainage surgery such as Ahmed glaucoma valve implants (New World Medical, Rancho Cucamonga, CA, USA), Molteno implants (Molteno Ophthalmic Limited, Dunedin, New Zealand), Baerveldt implants (Abbott Medical Optics, CA, USA), and EXPRESS shunts (Alcon, USA) and MIGS (subconjunctival devices, Schlemm's canal devices, suprachoroidal devices and cyclodestruction). Glaucoma laser treatments and peripheral iridotomy will be excluded.

Comparator(s)

We intend to compare interventions; glaucoma surgery alone or in combination with cataract surgery (phacoemulsification). The comparator could be no surgery or other type of glaucoma surgery (such as MIGS techniques or glaucoma filtration surgery) or phacoemulsification surgery.

Outcomes

This review will consider studies that include the following outcomes:

Primary outcome:

- Percentage change of corneal endothelial cell density at 1 year.

Secondary outcomes:

- Percentage change of corneal endothelial cell density at 2 years and 5 years.
- Mean post-operative corneal endothelial cell density (cell/mm2).
- Symptoms related to corneal endothelial cell density loss such as corneal edema, bullous keratopathy or requiring subsequent keratoplasty.
- Adverse events including but not limited to intra-operative complications and hyphema.

Types of studies

As randomized controlled trials (RCTs) with ECD data is limited for glaucoma surgery, we will evaluate the studies from a broad range of studies to have a comprehensive picture and maintain generalizability without loss of validity. These include RCTs, prospective non-randomized studies and observational studies. Studies published in English language will be included. Studies published from the year 2000 to the present will be included to maintain relevance as surgical techniques have progressed and improved in the recent years.

For peer review only

Methods

The proposed systematic review will be conducted in accordance with the Joanna Briggs Institute methodology for systematic reviews of effectiveness evidence.[19]

PROSPERO registration number: CRD42020192303

Search strategy

We will conduct a systematic electronic database search for randomized controlled trials, prospective non-randomized studies, observational studies in MEDLINE, EMBASE, Cochrane Central Register of Controlled Trials (CENTRAL), ClinicalTrials.gov and The International Prospective Register of Systematic Reviews (PROSPERO). The full search strategy with the keywords and index terms will be run on MEDLINE and EMBASE (see Appendix 1). The reference list of all studies selected will be screened for additional studies.

Study selection

Following the search, all identified citations will be collated and uploaded into a reference management software (Endnote X9, Clarivate Analytics) and duplicates will be removed. Two review authors will independently screen search results by title, abstract and keywords and then by full text, against the eligibility criteria, following the Preferred Reporting Items for Systematic Reviews and Meta-Analyses (PRISMA) guidelines.[20] Discrepancies between authors as to whether or not studies meet inclusion criteria will be resolved by discussion. We will document the excluded studies and reasons for exclusion, and this will be presented in a PRISMA flow diagram.[20]

Assessment of methodological quality

Eligible studies will be critically appraised by two independent reviewers at the study level using standardized critical appraisal instruments from the Joanna Briggs Institute.[21,22] Authors of papers will be contacted to request missing or additional data for clarification, where required. Any disagreements that arise between the reviewers will be resolved through discussion, or with a third reviewer. The results of critical appraisal will be reported in narrative form and in a table.

Two review authors will assess independently the risk of bias. Critical appraisal of study methodological rigor will be performed based on critical appraisal tools,[19,23,24] depending on the experimental design of the study being assessed. Any disagreements that arise between the reviewers will be resolved through discussion. Regardless of the results of their methodological quality, will undergo data extraction and synthesis. Forest plots will also be created to graphically depict the individual and pooled effect sizes. To assess risk of bias across all studies, funnel plots for each outcome will be assessed for symmetry (using Egger test, Begg test, Harbord test where appropriate) to determine if publication bias is present.

For peer review only - http://bmjopen.bmj.com/site/about/guidelines.xhtml

Data extraction

Data will be extracted from included studies by two independent reviewers aligned to the standardized data extraction tool recommended by JBI.[19,24] Variables to be extracted include:

- Study characteristics such as country of origin, year of publication and sample size
- Trial design
- Participant characteristics such as ages, gender, ethnicities
- Surgery type
- ECD pre and post-surgery and at assessment time points.
- Number of patients withdrawn from study
- Mean and standard deviation for each outcome

To minimize errors, a data extraction form has been developed for specifically this review (Appendix II). Any disagreements that arise between the reviewers will be resolved through discussion, or with a third reviewer. Authors of studies will be contacted to request additional or missing data, where required.

Data synthesis

Studies will, where possible, be pooled with statistical meta-analysis. Effect sizes will be expressed as either odds ratios (for dichotomous data) or weighted (or standardized) final post-intervention mean differences (for continuous data) and their 95% confidence intervals will be calculated for analysis. Heterogeneity will be assessed statistically using the standard chi squared and I^2 tests. Statistical analyses will be performed using RevMan 5.3. Subgroup analyses will be conducted where there is sufficient data to investigate ECD loss by type of surgery and glaucoma. Sensitivity analyses will be conducted to test decisions made regarding studies at high risk of bias for an outcome in one or more key domains; selection, performance, detection, attrition and reporting biases. Network meta-analysis will be conducted where appropriate to rank type of surgery according to ECD loss. Network diagram will graphically depict the distribution of available evidence for each comparison. This review will generate comparisons between different types of surgery on ECD loss in glaucoma patients. This will allow for comparison between surgeries that have not previously been compared. The network analysis based on a random effects model will be conservatively employed. Where statistical pooling is not possible the findings will be presented in narrative form including tables and figures to aid in data presentation, where appropriate.

Assessing certainty in the findings

The Grading of Recommendations, Assessment, Development and Evaluation (GRADE) approach for grading the certainty of evidence will be followed and a Summary of Findings (SoF) will be created using GRADEpro software (McMaster University, ON, Canada). We will grade the quality of evidence for each outcome by considering study limitations, indirectness, inconsistency, imprecision of effect estimates and risk of reporting bias. According to the software GRADEpro, we will assign four levels of quality of evidence: high, moderate, low and very low.

For peer review only - http://bmjopen.bmj.com/site/about/guidelines.xhtml

The SoF will present the following information where appropriate: absolute risks for the treatment and control, estimates of relative risk, and a ranking of the quality of the evidence based on the risk of bias, directness, heterogeneity, precision and risk of publication bias of the review results. The outcomes reported in the SoF will be presented in a tabular form. Outcomes for inclusion in SoF will be endothelial cell density loss.

We will present the main results of this review in a 'Summary of findings' (SoF) table, according to recommendations provided[23] in Chapter 11 of the Cochrane Handbook for Systematic Reviews of Interventions (version 5.1.0). We will provide estimates derived from the meta-analysis in accordance with methods of the GRADE (Grades of Recommendation, Assessment, Development and Evaluation) Working Group.[25]

For peer review only

For peer review only - http://bmjopen.bmj.com/site/about/guidelines.xhtml

1. Edelhauser HF. The balance between corneal transparency and edema: the Proctor Lecture. Invest Ophthalmol Vis Sci. 2006;47(5):1754-67.
2. Janson BJ, Alward WL, Kwon YH, Bettis DI, Fingert JH, Provencher LM, et al. Glaucoma-associated corneal endothelial cell damage: A review. Survey of Ophthalmology. 2018;63(4):500-6.
3. Sihota R, Lakshmaiah NC, Titiyal JS, Dada T, Agarwal HC. Corneal endothelial status in the subtypes of primary angle closure glaucoma. Clin Exp Ophthalmol. 2003;31(6):492-5.
4. Ward MS, Goins KM, Greiner MA, Kitzmann AS, Sutphin JE, Alward WLM, et al. Graft survival versus glaucoma treatment after penetrating or descemet stripping automated endothelial keratoplasty. Cornea. 2014;33(8):785-9.
5. Sugar A. Factors associated with corneal graft survival in the cornea donor study. JAMA Ophthalmology. 2015;133(3):246-54.
6. Gagnon MM, Boisjoly HM, Brunette I, Charest M, Amyot M. Corneal endothelial cell density in glaucoma. Cornea. 1997;16(3):314-8.
7. Yu ZY, Wu L, Qu B. Changes in corneal endothelial cell density in patients with primary open-angle glaucoma. World Journal of Clinical Cases. 2019;7(15):1978-85.
8. Hau S, Barton K. Corneal complications of glaucoma surgery. Current Opinion in Ophthalmology. 2009;20(2):131-6.
9. Christakis PG, Kalenak JW, Tsai JC, Zurakowski D, Kammer JA, Harasymowycz PJ, et al. The Ahmed Versus Baerveldt Study: Five-Year Treatment Outcomes. Ophthalmology. 2016;123(10):2093-102.
10. Budenz DL, Barton K, Gedde SJ, Feuer WJ, Schiffman J, Costa VP, et al. Five-year treatment outcomes in the Ahmed Baerveldt comparison study. Ophthalmology. 2015;122(2):308-16.
11. Lesiewska-Junk H, Kałuzny J, Malukiewicz-Wiśniewska G. Long-term evaluation of endothelial cell loss after phacoemulsification. Eur J Ophthalmol. 2002;12(1):30-3.
12. Bourne WM, Nelson LR, Hodge DO. Continued endothelial cell loss ten years after lens implantation. Ophthalmology. 1994;101(6):1014-22; discussion 22-3.
13. Alcon Research, LTD. Recalls CyPass Micro-Stent Systems Due to Risk of Endothelial Cell Loss: FDA; 2018 [Available from: https://www.fda.gov/medical-devices/medical-device-recalls/alcon-research-ltd-recalls-cypassr-micro-stent-systems-due-risk-endothelial-cell-los.
14. Lass JH, Benetz BA, He J, Hamilton C, Von Tress M, Dickerson J, et al. Corneal Endothelial Cell Loss and Morphometric Changes 5 Years after Phacoemulsification with or without CyPass Micro-Stent. American Journal of Ophthalmology. 2019;208:211-8.
15. Vold S, Ahmed, II, Craven ER, Mattox C, Stamper R, Packer M, et al. Two-Year COMPASS Trial Results: Supraciliary Microstenting with Phacoemulsification in Patients with Open-Angle Glaucoma and Cataracts. Ophthalmology. 2016;123(10):2103-12.
16. Can İ, Bayhan HA, Çelik H, Ceran BB. Comparison of corneal aberrations after biaxial microincision and microcoaxial cataract surgeries: a prospective study. Curr Eye Res. 2012;37(1):18-24.
17. Dosso AA, Cottet L, Burgener ND, Di Nardo S. Outcomes of coaxial microincision cataract surgery versus conventional coaxial cataract surgery. J Cataract Refract Surg. 2008;34(2):284-8.

For peer review only

For peer review only - http://bmjopen.bmj.com/site/about/guidelines.xhtml

18. Dada T, Bhartiya S, Begum Baig N. Cataract Surgery in Eyes with Previous Glaucoma Surgery: Pearls and Pitfalls. Journal of current glaucoma practice. 2013;7(3):99-105.
19. Peters MD, Godfrey CM, Khalil H, McInerney P, Parker D, Soares CB. Guidance for conducting systematic scoping reviews. Int J Evid Based Healthc. 2015;13(3):141-6.
20. Moher D, Liberati A, Tetzlaff J, Altman DG. Preferred reporting items for systematic reviews and meta-analyses: the PRISMA statement. BMJ. 2009;339:b2535.
21. Moola S MZ, Tufanaru C, Aromataris E, Sears K, Sfetcu R, Currie M, Qureshi R, Mattis P, Lisy K, Mu P-F. Chapter 7: Systematic reviews of etiology and risk The Joanna Briggs Institute2017 [Available from: https://reviewersmanual.joannabriggs.org.
22. Tufanaru C MZ, Aromataris E, Campbell J, Hopp L. . Chapter 3: Systematic reviews of effectiveness. Joanna Briggs Institute Reviewer's Manual: The Joanna Briggs Institute; 2017 [Available from: https://reviewersmanual.joannabriggs.org/.
23. Higgins JP, Altman DG, Gøtzsche PC, Jüni P, Moher D, Oxman AD, et al. The Cochrane Collaboration's tool for assessing risk of bias in randomised trials. Bmj. 2011;343:d5928.
24. Aromataris E FR, Godfrey C, Holly C, Kahlil H, Tungpunkom P. Summarizing systematic reviews: methodological development, conduct and reporting of an Umbrella review approach Joanna Briggs Institute Reviewer's Manual 2017 [Available from: https://reviewersmanual.joannabriggs.org/.
25. Atkins D, Best D, Briss PA, Eccles M, Falck-Ytter Y, Flottorp S, et al. Grading quality of evidence and strength of recommendations. Bmj. 2004;328(7454):1490.

For peer review only

Appendix I: Search strategy

EMBASE

Search conducted in May 2020

Search	Query	Records Retrieved
1	exp glaucoma/	77252
2	glaucoma*.mp.	84904
3	1 or 2	88723
4	exp eye surgery/ or exp surgery/	4584292
5	(Surg* or procedure* or operat* or incision* or invasive* or penetrat* or Drainage implant surg* or Electrocautery or Cyclophotocoagulation*).mp.	6437635
6	4 or 5	7735880
7	endothelial* cell*.ab. or endothelial* cell*.ti.	214675
8	3 and 6 and 7	870
9	exp cataract/	54959
10	exp phacoemulsification/	14524

For peer review only

11	(cataract* or pha?oemulsif*).mp. [mp=title, abstract, heading word, drug trade name, original title, device manufacturer, drug manufacturer, device trade name, keyword, floating subheading word, candidate term word]	91182
12	9 or 10 or 11	92259
13	exp eye surgery/ or exp surgery/	4584292
14	(Surg* or procedure* or operat* or incision*).mp.	6019212
15	13 or 14	7359674
16	(micro or small or minimally or minimal).mp.	2452911
17	(microincision* or micro-incision* or incision*).ti.	18429
18	16 or 17	2465927
19	7 and 12 and 15 and 18	315
20	8 or 19	1138
21	limit 20 to (human and english language and yr="2000 -Current")	724

MEDLINE

Search conducted in May 2020

Search	Query	Records Retrieved

For peer review only

1	exp Glaucoma, Angle-Closure/ or exp Low Tension Glaucoma/ or exp Glaucoma/ or exp Glaucoma, Open-Angle/ or exp Glaucoma, Neovascular/ or exp Glaucoma Drainage Implants/	52351
2	glaucoma*.mp.	70177
3	1 or 2	70398
4	exp General Surgery/	38657
5	(Surg* or procedure* or operat* or incision* or invasive* or penetrat* or Drainage implant surg* or Electrocautery or Cyclophotocoagulation*).mp.	4681137
6	4 or 5	4681137
7	exp Endothelial Cells/	58726
8	endothelial* cell*.ab. or endothelial* cell*.ti.	164910
9	7 or 8	178945
10	3 and 6 and 9	509
11	exp Cataract/	28781
12	exp Phacoemulsification/	9762
13	(cataract* or pha?oemulsif*).mp.	72591
14	11 or 12 or 13	72661

For peer review only

15	exp General Surgery/	38657
16	(Surg* or procedure* or operat* or incision*).mp.	4326472
17	15 or 16	4326472
18	(micro or small or minimally or minimal).mp.	1918877
19	(incision* or (microincision* or micro-incision*)).ti.	15328
20	18 or 19	1930443
21	9 and 14 and 17 and 20	218
22	10 or 21	708
23	limit 22 to (english language and humans and yr="2000 -Current")	408

Appendix II: Draft data extraction form

Reviewer name:	
**Citation details:**<br>Authors:<br>Title:<br>Journal:<br>Year:	
**Study details:**<br>Study design:<br>Sample size:<br>Country:	
**Participant characteristics:**<br>Age:<br>Sex:	

For peer review only

**Inclusion criteria**<br>**Exclusion criteria**	
**Surgery type:**<br>**Intervention:**<br>**Comparator(s):**	
**Outcome**: (at all time assessment points)<br>Mean ECD pre-operation:<br>Mean ECD post-operation:<br>Percentage ECD loss:<br>Corneal complications/adverse events:	

For peer review only

PRISMA-P 2015 Checklist

This checklist has been adapted for use with protocol submissions to *Systematic Reviews* from Table 3 in Moher D et al: Preferred reporting items for systematic review and meta-analysis protocols (PRISMA-P) 2015 statement. *Systematic Reviews* 2015 **4**:1

Section/topic	#	Checklist item	Information reported Yes	No	Line number(s)
**ADMINISTRATIVE INFORMATION**					
**Title**					
Identification	1a	Identify the report as a protocol of a systematic review	☒	☐	5-14
Update	1b	If the protocol is for an update of a previous systematic review, identify as such	☐	☐	NA
**Registration**	2	If registered, provide the name of the registry (e.g., PROSPERO) and registration number in the Abstract	☒	☐	82
**Authors**					
Contact	3a	Provide name, institutional affiliation, and e-mail address of all protocol authors; provide physical mailing address of corresponding author	☒	☐	39-43
Contributions	3b	Describe contributions of protocol authors and identify the guarantor of the review	☒	☐	24-27
**Amendments**	4	If the protocol represents an amendment of a previously completed or published protocol, identify as such and list changes; otherwise, state plan for documenting important protocol amendments	☐	☐	NA
**Support**					
Sources	5a	Indicate sources of financial or other support for the review	☒	☐	34-39
Sponsor	5b	Provide name for the review funder and/or sponsor	☒	☐	34-39
Role of sponsor/funder	5c	Describe roles of funder(s), sponsor(s), and/or institution(s), if any, in developing the protocol	☐	☐	NA
**INTRODUCTION**					
**Rationale**	6	Describe the rationale for the review in the context of what is already known	☒	☐	87-144
**Objectives**	7	Provide an explicit statement of the question(s) the review will address with reference to participants, interventions, comparators, and outcomes (PICO)	☒	☐	141-144
**METHODS**					

BioMed Central
The Open Access Publisher

Section/topic	#	Checklist item	Information reported Yes	No	Line number(s)
**Eligibility criteria**	8	Specify the study characteristics (e.g., PICO, study design, setting, time frame) and report characteristics (e.g., years considered, language, publication status) to be used as criteria for eligibility for the review	☒	☐	145-182
**Information sources**	9	Describe all intended information sources (e.g., electronic databases, contact with study authors, trial registers, or other grey literature sources) with planned dates of coverage	☒	☐	183-194
**Search strategy**	10	Present draft of search strategy to be used for at least one electronic database, including planned limits, such that it could be repeated	☒	☐	183-194
STUDY RECORDS					
Data management	11a	Describe the mechanism(s) that will be used to manage records and data throughout the review	☒	☐	195-262
Selection process	11b	State the process that will be used for selecting studies (e.g., two independent reviewers) through each phase of the review (i.e., screening, eligibility, and inclusion in meta-analysis)	☒	☐	195-262
Data collection process	11c	Describe planned method of extracting data from reports (e.g., piloting forms, done independently, in duplicate), any processes for obtaining and confirming data from investigators	☒	☐	195-262
**Data items**	12	List and define all variables for which data will be sought (e.g., PICO items, funding sources), any pre-planned data assumptions and simplifications	☒	☐	217-229
**Outcomes and prioritization**	13	List and define all outcomes for which data will be sought, including prioritization of main and additional outcomes, with rationale	☒	☐	164-174
**Risk of bias in individual studies**	14	Describe anticipated methods for assessing risk of bias of individual studies, including whether this will be done at the outcome or study level, or both; state how this information will be used in data synthesis	☒	☐	203 -216
DATA					
**Synthesis**	15a	Describe criteria under which study data will be quantitatively synthesized	☒	☐	230-240
	15b	If data are appropriate for quantitative synthesis, describe planned summary measures, methods of handling data, and methods of combining data from studies, including any planned exploration of consistency (e.g., I^2, Kendall's tau)	☒	☐	230-240
	15c	Describe any proposed additional analyses (e.g., sensitivity or subgroup analyses, meta-regression)	☒	☐	230-240
	15d	If quantitative synthesis is not appropriate, describe the type of summary planned	☒	☐	230-240
**Meta-bias(es)**	16	Specify any planned assessment of meta-bias(es) (e.g., publication bias across studies, selective reporting within studies)	☒	☐	246-252
**Confidence in cumulative evidence**	17	Describe how the strength of the body of evidence will be assessed (e.g., GRADE)	☒	☐	246-262

For peer review only

BioMed Central The Open Access Publisher

BMJ Open

Corneal endothelial cell density loss following glaucoma surgery alone or in combination with cataract surgery: A systematic review protocol

Journal:	*BMJ Open*
Manuscript ID	bmjopen-2021-050992.R1
Article Type:	Protocol
Date Submitted by the Author:	15-Aug-2021

Complete List of Authors:	Fang, Clarissa E H; University College London Institute of Ophthalmology<br>Khaw, Peng; University College London Institute of Ophthalmology; NIHR Moorfields Biomedical Research Centre<br>Mathew, Rashmi G; University College London Institute of Ophthalmology; Moorfields Eye Hospital NHS Foundation Trust<br>Henein, Christin; University College London Institute of Ophthalmology; NIHR Moorfields Biomedical Research Centre

**Primary Subject Heading**:	Ophthalmology
Secondary Subject Heading:	Surgery
Keywords:	Glaucoma < OPHTHALMOLOGY, Cataract and refractive surgery < OPHTHALMOLOGY, Ophthalmology < SURGERY

SCHOLARONE™
Manuscripts

I, the Submitting Author has the right to grant and does grant on behalf of all authors of the Work (as defined in the below author licence), an exclusive licence and/or a non-exclusive licence for contributions from authors who are: i) UK Crown employees; ii) where BMJ has agreed a CC-BY licence shall apply, and/or iii) in accordance with the terms applicable for US Federal Government officers or employees acting as part of their official duties; on a worldwide, perpetual, irrevocable, royalty-free basis to BMJ Publishing Group Ltd ("BMJ") its licensees and where the relevant Journal is co-owned by BMJ to the co-owners of the Journal, to publish the Work in this journal and any other BMJ products and to exploit all rights, as set out in our licence.

The Submitting Author accepts and understands that any supply made under these terms is made by BMJ to the Submitting Author unless you are acting as an employee on behalf of your employer or a postgraduate student of an affiliated institution which is paying any applicable article publishing charge ("APC") for Open Access articles. Where the Submitting Author wishes to make the Work available on an Open Access basis (and intends to pay the relevant APC), the terms of reuse of such Open Access shall be governed by a Creative Commons licence – details of these licences and which Creative Commons licence will apply to this Work are set out in our licence referred to above.

Other than as permitted in any relevant BMJ Author's Self Archiving Policies, I confirm this Work has not been accepted for publication elsewhere, is not being considered for publication elsewhere and does not duplicate material already published. I confirm all authors consent to publication of this Work and authorise the granting of this licence.

For peer review only

Title

Corneal endothelial cell density loss following glaucoma surgery alone or in combination with cataract surgery: A systematic review protocol

Authors

Clarissa E H Fang[1], Peng T Khaw[1,2], Rashmi G Mathew[1,3], Christin Henein[1,2]

Affiliations

[1]UCL, Institute of Ophthalmology, London, UK
[2] NIHR Moorfields Biomedical Research Centre, London, UK
[3] Moorfields Eye Hospital NHS Foundation Trust, London, UK

Corresponding author
Christin Henein
c.henein@ucl.ac.uk

I, the Submitting Author has the right to grant and does grant on behalf of all authors of the Work (as defined in the below author licence), an exclusive licence and/or a non-exclusive licence for contributions from authors who are: i) UK Crown employees; ii) where BMJ has agreed a CC-BY licence shall apply, and/or iii) in accordance with the terms applicable for US Federal Government officers or employees acting as part of their official duties; on a worldwide, perpetual, irrevocable, royalty-free basis to BMJ Publishing Group Ltd ("BMJ") its licensees and where the relevant Journal is co-owned by BMJ to the co-owners of the Journal, to publish the Work in BMJ Open and any other BMJ products and to exploit all rights, as set out in our licence.

Data statement

Supplementary materials, appendix, and dataset will be made available in a data repository.

Patient and Public Involvement

Patients and public were not involved in the development of this protocol. However, the James Lind Alliance has prioritised the need to identify safe and effective treatments for glaucoma.

Email addresses:

For peer review only

Clarissa E H Fang fangclarissa@gmail.com

Peng T Khaw p.khaw@ucl.ac.uk

Rashmi G Mathew r.mathew@ucl.ac.uk

Christin Henein c.henein@ucl.ac.uk

For peer review only

Abstract

Objective: We aim to systematically assess and compare corneal endothelial cell density (ECD) loss in patients with glaucoma following glaucoma surgery and cataract surgery.

Introduction: Corneal ECD loss may occur due to intraoperative surgical trauma in glaucoma surgery or postoperatively with chronic endothelial cell trauma or irritation. Corneal oedema and decompensation after aqueous shunt glaucoma surgery has been reported but the long term ECD loss is still unknown.

Inclusion criteria: Trabeculectomy, glaucoma filtration surgery or microinvasive glaucoma surgery in adults with ocular hypertension, primary and secondary open angle glaucoma, normal tension glaucoma and angle-closure glaucoma. Participants with pre-existing corneal disease will be excluded. Glaucoma laser treatments and peripheral iridotomy will be excluded. The outcomes include preoperative and postoperative corneal ECD, percentage change of corneal ECD and adverse events.

Methods: We will conduct an electronic database search for randomized controlled trials, prospective non-randomized studies, observational studies in MEDLINE, EMBASE, Cochrane Central Register of Controlled Trials (CENTRAL), ClinicalTrials.gov and The International Prospective Register of Systematic Reviews (PROSPERO). Eligibility criteria will include quantitative articles published after and including the year 2000, written in English and containing data on ECD loss. Two independent reviewers will screen titles and abstracts and extract data from full texts, reporting outcomes according to Preferred Reporting Items for Systematic Reviews and Meta-Analyses (PRISMA) guidelines. Data extraction of key characteristics will be completed using customized forms. Methodological quality will be assessed using the Joanna Briggs Institute critical appraisal forms.

Ethics and dissemination: Ethics approval is not required for this review, as it will only include published data. Findings will be published in a peer-reviewed journal and disseminated across ophthalmic networks.

Systematic review registration number: PROSPERO CRD42020192303

Keywords: glaucoma surgery, cataract surgery, endothelial cell density, minimally invasive glaucoma surgery, trabeculectomy

Article Summary

Strengths and Limitations

- This protocol adheres to the Preferred Reporting Items for Systematic Reviews and Meta-Analysis protocols and has been published prospectively in PROSPERO database

For peer review only

- The review will comprehensively assess published peer-reviewed manuscripts assessing endothelial cell loss and adverse events following glaucoma surgery
- A potential limitation might be the paucity of high-quality glaucoma surgery trials measuring endothelial cell density loss
- Due to the expected heterogeneity in study methods, it is unlikely that a network meta-analysis will be possible

Word count: 2112 words

Introduction

The corneal endothelium is a monolayer of hexagonal cells lining the posterior corneal surface that regulates stromal hydration. This squamous cell layer does not regenerate and if damaged, the cornea becomes oedematous, causing loss of corneal clarity thus compromising vision, negatively affecting quality of life. Physiologically endothelial cell density (ECD) reduces with age at a rate 0.5% per year.[1] Glaucoma is a known condition that predisposes to endothelial cell damage, although the underlying mechanisms are not well elucidated.[2] Certain types of glaucoma are more predisposed to ECD loss such as when there is a sharp rise in intraocular pressure (IOP) in acute angle closure.[3] Glaucoma has also been associated with poor cornea graft survival.[4,5] High levels of IOP as well as long duration of high IOP are associated with more ECD loss.[2,6,7] It remains inconclusive whether glaucoma medications cause endothelial cell loss.[2]

Glaucoma surgery is effective in reducing IOP and preventing the progression of optic nerve damage and visual field loss. Trabeculectomy, or bleb filtration surgery, is considered the gold standard and is frequently performed for medically uncontrolled glaucoma. Aqueous drainage shunts have conventionally been used in cases of uncontrolled glaucoma following unsuccessful trabeculectomy. However, some corneal endothelial cell damage after surgery is inevitable. In the literature, corneal oedema after aqueous shunt surgery due to corneal decompensation has been reported.[8] Large randomized controlled trials on aqueous shunt surgery; The Ahmed vs Baerveldt Study and Ahmed Baerveldt Comparison Study had an average incidence rate of 12% persistent corneal oedema 5 years post-operation, but did not measure or report ECD loss.[9,10] The safety of glaucoma filtration surgery has progressed with the advancement from full-thickness procedures to ab interno surgery and more recently, microinvasive glaucoma surgery (MIGS). MIGS is frequently combined with cataract surgery which in itself is known to cause ECD loss.[11,12] Implantable devices used in glaucoma surgery are manufactured from different materials with varying biocompatibility profiles. The location of implants and their proximity from endothelial cells, tube stability and micromovements can impact ECD. Recently, there has been a growing use of new ophthalmic implants for treatment of glaucoma. ECD loss is an important surrogate marker for device safety and surgical failure. It is used as a requirement for some Food and Drug Administration (FDA) approved investigational device exemption (IDE) trials and an endpoint for post-market approval surveillance studies. In August 2018,

For peer review only - http://bmjopen.bmj.com/site/about/guidelines.xhtml

the MIGS device Cypass Micro-Stent (Alcon, USA) was voluntarily withdrawn from the market due to safety concerns when it was reported that 27.2% of patients had ECD loss of >30% at 5 years.[13,14] Interestingly, the device was not considered to have continuing ECD loss at 2 years in a previous study.[15]

The common cataract surgery method of phacoemulsification is where ultrasound is used to break down and aspirate the cataractous lens in the eye of a patient, followed by insertion of an intraocular lens. Cataract surgery has evolved in the last decade with new microincisional techniques which aim to cause less damage to the cornea.[16,17] This pertains to glaucoma patients requiring cataract surgery as well.[18] Patients with glaucoma often have co-existing cataracts and will eventually require surgery for one or the other. It is possible to undergo glaucoma surgery and cataract surgery in the same operation. MIGS is frequently combined with cataract surgery. In fact, in the United states, some stents used in Schlemm's canal are only licensed for combination surgery and not for stand-alone procedures. This is reflected in trials where the intervention is combination surgery compared to phacoemulsification alone.[14,15] Combination surgery carries a distinctive risk-benefit evaluation compared to glaucoma surgery alone. Theoretically, further cost and risks of adding a short procedure to an existing operation may be less than those acquired by a separate hospital admission and anaesthetic. These combination surgeries may consequently promote a lower threshold for the use of MIGS in clinical practice. However, combination surgery can negatively affect surgical outcome in complicated cases. Since cataract surgery also causes ECD loss, it is an important confounder in many glaucoma surgery trials.

This review focuses on ECD loss from trabeculectomy, glaucoma filtration surgery and MIGS, alone or in combination with cataract surgery. A preliminary search of PROSPERO, MEDLINE, the Cochrane Database of Systematic Reviews and the *JBI Database of Systematic Reviews and Implementation Reports* was conducted and no current or underway systematic reviews on the topic were identified. There is a need to quantitatively and critically appraise available evidence, and to evaluate where gaps exist.

Review question

To determine the extent of endothelial cell density loss following different types of glaucoma surgery alone or in combination with cataract surgery in adults with ocular hypertension, primary and secondary open angle glaucoma, normal tension glaucoma and angle-closure glaucoma.

Inclusion criteria

Participants

This review will consider studies that include participants with ocular hypertension, primary and secondary open angle glaucoma, normal tension glaucoma and angle-closure glaucoma. There are no restrictions on geographic location, setting or demographic factors. Participants with pre-existing

For peer review only

corneal disease such as Fuch's endothelial dystrophy, iridocorneal endothelial syndrome and corneal graft transplant and the paediatric population will be excluded.

Intervention(s)

We will include incisional glaucoma surgery such as trabeculectomy, glaucoma drainage surgery such as Ahmed glaucoma valve implants (New World Medical, Rancho Cucamonga, CA, USA), Molteno implants (Molteno Ophthalmic Limited, Dunedin, New Zealand), Baerveldt implants (Abbott Medical Optics, CA, USA), and EXPRESS shunts (Alcon, USA) and MIGS (subconjunctival devices, Schlemm's canal devices, suprachoroidal devices and cyclodestruction). Glaucoma laser treatments and peripheral iridotomy will be excluded.

Comparator(s)

We intend to compare interventions; glaucoma surgery alone or in combination with cataract surgery (phacoemulsification). The comparator could be no surgery or other type of glaucoma surgery (such as MIGS techniques or glaucoma filtration surgery) or phacoemulsification surgery.

Outcomes

This review will consider studies that include the following outcomes:

Primary outcome:

- Percentage change of corneal endothelial cell density at 1 year.

Secondary outcomes:

- Percentage change of corneal endothelial cell density at 2 years and 5 years.
- Mean post-operative corneal endothelial cell density (cell/mm2).
- Symptoms related to corneal endothelial cell density loss such as corneal oedema, bullous keratopathy or requiring subsequent keratoplasty.
- Adverse events including but not limited to intra-operative complications and hyphaema.

Types of studies

As randomized controlled trials (RCTs) with ECD data is limited for glaucoma surgery, we will evaluate the studies from a broad range of studies to have a comprehensive picture and maintain generalizability without loss of validity. These include RCTs, prospective non-randomized studies and observational studies. Studies published in English language will be included. Studies published from the year 2000 to the present will be included to maintain relevance as surgical techniques have progressed and improved in the recent years.

Methods and analysis

For peer review only

The proposed systematic review will be conducted in accordance with the Joanna Briggs Institute methodology for systematic reviews of effectiveness evidence.[19] Study start date June 2020 and anticipated end date June 2022.

PROSPERO registration number: CRD42020192303

Search strategy

We will conduct a systematic electronic database search for randomized controlled trials, prospective non-randomized studies, observational studies in MEDLINE, EMBASE, Cochrane Central Register of Controlled Trials (CENTRAL), ClinicalTrials.gov and The International Prospective Register of Systematic Reviews (PROSPERO). The full search strategy with the keywords and index terms will be run on MEDLINE and EMBASE (see Appendix 1). The reference list of all studies selected will be screened for additional studies.

Study selection

Following the search, all identified citations will be collated and uploaded into a reference management software (Endnote X9, Clarivate Analytics) and duplicates will be removed. Two review authors will independently screen search results by title, abstract and keywords and then by full text, against the eligibility criteria, following the Preferred Reporting Items for Systematic Reviews and Meta-Analyses (PRISMA) guidelines.[20] Discrepancies between authors as to whether or not studies meet inclusion criteria will be resolved by discussion. We will document the excluded studies and reasons for exclusion, and this will be presented in a PRISMA flow diagram.[20]

Assessment of methodological quality

Eligible studies will be critically appraised by two independent reviewers at the study level using standardized critical appraisal instruments from the Joanna Briggs Institute.[21,22] Authors of papers will be contacted to request missing or additional data for clarification, where required. Any disagreements that arise between the reviewers will be resolved through discussion, or with a third reviewer. The results of critical appraisal will be reported in narrative form and in a table.

Two review authors will assess independently the risk of bias. Critical appraisal of study methodological rigor will be performed based on critical appraisal tools,[19,23,24] depending on the experimental design of the study being assessed. Any disagreements that arise between the reviewers will be resolved through discussion. Regardless of the results of their methodological quality, will undergo data extraction and synthesis. Forest plots will also be created to graphically depict the individual and pooled effect sizes. To assess risk of bias across all studies, funnel plots for each outcome will be assessed for symmetry (using Egger test, Begg test, Harbord test where appropriate) to determine if publication bias is present.

For peer review only - http://bmjopen.bmj.com/site/about/guidelines.xhtml

Data extraction

Data will be extracted from included studies by two independent reviewers aligned to the standardized data extraction tool recommended by JBI.[19,24] Variables to be extracted include:

- Study characteristics such as country of origin, year of publication and sample size
- Trial design
- Participant characteristics such as ages, gender, ethnicities
- Glaucoma type
- Surgery type and site of implant
- ECD pre and post-surgery and at assessment time points.
- Number of patients withdrawn from study
- Mean and standard deviation for each outcome

To minimize errors, a data extraction form has been developed for specifically this review (Appendix II). Any disagreements that arise between the reviewers will be resolved through discussion, or with a third reviewer. Authors of studies will be contacted to request additional or missing data, where required.

Data synthesis

Studies will, where possible, be pooled with statistical meta-analysis. Effect sizes will be expressed as either odds ratios (for dichotomous data) or weighted (or standardized) final post-intervention mean differences (for continuous data) and their 95% confidence intervals will be calculated for analysis. Heterogeneity will be assessed statistically using the standard chi squared and I^2 tests. Statistical analyses will be performed using RevMan 5.3. Subgroup analyses will be conducted where there is sufficient data to investigate ECD loss by type of surgery and glaucoma. Sensitivity analyses will be conducted to test decisions made regarding studies at high risk of bias for an outcome in one or more key domains; selection, performance, detection, attrition and reporting biases. Network meta-analysis will be conducted where appropriate to rank type of surgery according to ECD loss. Network diagram will graphically depict the distribution of available evidence for each comparison. This review will generate comparisons between different types of surgery on ECD loss in glaucoma patients. This will allow for comparison between surgeries that have not previously been compared. The network analysis based on a random effects model will be conservatively employed. Where statistical pooling is not possible the findings will be presented in narrative form including tables and figures to aid in data presentation, where appropriate.

Assessing certainty in the findings

The Grading of Recommendations, Assessment, Development and Evaluation (GRADE) approach for grading the certainty of evidence will be followed and a Summary of Findings (SoF) will be created using GRADEpro software (McMaster University, ON, Canada). We will grade the quality of evidence for each outcome by considering study limitations, indirectness, inconsistency, imprecision of effect

For peer review only - http://bmjopen.bmj.com/site/about/guidelines.xhtml

estimates and risk of reporting bias. According to the software GRADEpro, we will assign four levels of quality of evidence: high, moderate, low and very low.

The SoF will present the following information where appropriate: absolute risks for the treatment and control, estimates of relative risk, and a ranking of the quality of the evidence based on the risk of bias, directness, heterogeneity, precision and risk of publication bias of the review results. The outcomes reported in the SoF will be presented in a tabular form. Outcomes for inclusion in SoF will be endothelial cell density loss.

We will present the main results of this review in a 'Summary of findings' (SoF) table, according to recommendations provided[23] in Chapter 11 of the Cochrane Handbook for Systematic Reviews of Interventions (version 5.1.0). We will provide estimates derived from the meta-analysis in accordance with methods of the GRADE (Grades of Recommendation, Assessment, Development and Evaluation) Working Group.[25]

Ethics and Dissemination

Ethics approval is not required for this review, as it will only include published data. Findings will be published in a peer-reviewed journal and disseminated across ophthalmic networks. We anticipate that the findings of this work will be of interest to multiple stakeholders: people who have undergone glaucoma surgery, eye health professionals, ophthalmic surgeons, device manufacturers and policy makers. It will also inform researchers to where there are gaps in evidence and identify areas for future research.

For peer review only

1. Edelhauser HF. The balance between corneal transparency and oedema: the Proctor Lecture. Invest Ophthalmol Vis Sci. 2006;47(5):1754-67.
2. Janson BJ, Alward WL, Kwon YH, Bettis DI, Fingert JH, Provencher LM, et al. Glaucoma-associated corneal endothelial cell damage: A review. Survey of Ophthalmology. 2018;63(4):500-6.
3. Sihota R, Lakshmaiah NC, Titiyal JS, Dada T, Agarwal HC. Corneal endothelial status in the subtypes of primary angle closure glaucoma. Clin Exp Ophthalmol. 2003;31(6):492-5.
4. Ward MS, Goins KM, Greiner MA, Kitzmann AS, Sutphin JE, Alward WLM, et al. Graft survival versus glaucoma treatment after penetrating or descemet stripping automated endothelial keratoplasty. Cornea. 2014;33(8):785-9.
5. Sugar A. Factors associated with corneal graft survival in the cornea donor study. JAMA Ophthalmology. 2015;133(3):246-54.
6. Gagnon MM, Boisjoly HM, Brunette I, Charest M, Amyot M. Corneal endothelial cell density in glaucoma. Cornea. 1997;16(3):314-8.
7. Yu ZY, Wu L, Qu B. Changes in corneal endothelial cell density in patients with primary open-angle glaucoma. World Journal of Clinical Cases. 2019;7(15):1978-85.
8. Hau S, Barton K. Corneal complications of glaucoma surgery. Current Opinion in Ophthalmology. 2009;20(2):131-6.
9. Christakis PG, Kalenak JW, Tsai JC, Zurakowski D, Kammer JA, Harasymowycz PJ, et al. The Ahmed Versus Baerveldt Study: Five-Year Treatment Outcomes. Ophthalmology. 2016;123(10):2093-102.
10. Budenz DL, Barton K, Gedde SJ, Feuer WJ, Schiffman J, Costa VP, et al. Five-year treatment outcomes in the Ahmed Baerveldt comparison study. Ophthalmology. 2015;122(2):308-16.
11. Lesiewska-Junk H, Kałuzny J, Malukiewicz-Wiśniewska G. Long-term evaluation of endothelial cell loss after phacoemulsification. Eur J Ophthalmol. 2002;12(1):30-3.
12. Bourne WM, Nelson LR, Hodge DO. Continued endothelial cell loss ten years after lens implantation. Ophthalmology. 1994;101(6):1014-22; discussion 22-3.
13. Alcon Research, LTD. Recalls CyPass Micro-Stent Systems Due to Risk of Endothelial Cell Loss: FDA; 2018 [Available from: https://www.fda.gov/medical-devices/medical-device-recalls/alcon-research-ltd-recalls-cypassr-micro-stent-systems-due-risk-endothelial-cell-los.
14. Lass JH, Benetz BA, He J, Hamilton C, Von Tress M, Dickerson J, et al. Corneal Endothelial Cell Loss and Morphometric Changes 5 Years after Phacoemulsification with or without CyPass Micro-Stent. American Journal of Ophthalmology. 2019;208:211-8.
15. Vold S, Ahmed, II, Craven ER, Mattox C, Stamper R, Packer M, et al. Two-Year COMPASS Trial Results: Supraciliary Microstenting with Phacoemulsification in Patients with Open-Angle Glaucoma and Cataracts. Ophthalmology. 2016;123(10):2103-12.
16. Can İ, Bayhan HA, Çelik H, Ceran BB. Comparison of corneal aberrations after biaxial microincision and microcoaxial cataract surgeries: a prospective study. Curr Eye Res. 2012;37(1):18-24.
17. Dosso AA, Cottet L, Burgener ND, Di Nardo S. Outcomes of coaxial microincision cataract surgery versus conventional coaxial cataract surgery. J Cataract Refract Surg. 2008;34(2):284-8.

For peer review only

For peer review only - http://bmjopen.bmj.com/site/about/guidelines.xhtml

18. Dada T, Bhartiya S, Begum Baig N. Cataract Surgery in Eyes with Previous Glaucoma Surgery: Pearls and Pitfalls. Journal of current glaucoma practice. 2013;7(3):99-105.
19. Peters MD, Godfrey CM, Khalil H, McInerney P, Parker D, Soares CB. Guidance for conducting systematic scoping reviews. Int J Evid Based Healthc. 2015;13(3):141-6.
20. Moher D, Liberati A, Tetzlaff J, Altman DG. Preferred reporting items for systematic reviews and meta-analyses: the PRISMA statement. BMJ. 2009;339:b2535.
21. Moola S MZ, Tufanaru C, Aromataris E, Sears K, Sfetcu R, Currie M, Qureshi R, Mattis P, Lisy K, Mu P-F. Chapter 7: Systematic reviews of etiology and risk The Joanna Briggs Institute2017 [Available from: https://reviewersmanual.joannabriggs.org.
22. Tufanaru C MZ, Aromataris E, Campbell J, Hopp L. . Chapter 3: Systematic reviews of effectiveness. Joanna Briggs Institute Reviewer's Manual: The Joanna Briggs Institute; 2017 [Available from: https://reviewersmanual.joannabriggs.org/.
23. Higgins JP, Altman DG, Gøtzsche PC, Jüni P, Moher D, Oxman AD, et al. The Cochrane Collaboration's tool for assessing risk of bias in randomised trials. Bmj. 2011;343:d5928.
24. Aromataris E FR, Godfrey C, Holly C, Kahlil H, Tungpunkom P. Summarizing systematic reviews: methodological development, conduct and reporting of an Umbrella review approach Joanna Briggs Institute Reviewer's Manual 2017 [Available from: https://reviewersmanual.joannabriggs.org/.
25. Atkins D, Best D, Briss PA, Eccles M, Falck-Ytter Y, Flottorp S, et al. Grading quality of evidence and strength of recommendations. Bmj. 2004;328(7454):1490.

Authors contribution

CH conceived the idea for the review. CEHF and CH drafted and revised the protocol with suggestions from PTK and RGM who reviewed the protocol and provided feedback on the draft. CEHF constructed the search.

Conflicts of interest

The authors declare no conflict of interest.

Acknowledgments

The authors would like to acknowledge the funding as mentioned below.

Funding

The authors receive funding from the National Institute for Health Research Biomedical Research Centre at Moorfields Eye Hospital NHS Foundation Trust and UCL Institute of Ophthalmology, the UK

For peer review only

Medical Research Council, Moorfields Eye Charity, the Michael and Ilse Katz Foundation, the Helen Hamlyn Trust and Fight for Sight (UK).

For peer review only

Appendix I: Search strategy

EMBASE

Search conducted in May 2020

Search	Query	Records Retrieved
1	exp glaucoma/	77252
2	glaucoma*.mp.	84904
3	1 or 2	88723
4	exp eye surgery/ or exp surgery/	4584292
5	(Surg* or procedure* or operat* or incision* or invasive* or penetrat* or Drainage implant surg* or Electrocautery or Cyclophotocoagulation*).mp.	6437635
6	4 or 5	7735880
7	endothelial* cell*.ab. or endothelial* cell*.ti.	214675
8	3 and 6 and 7	870
9	exp cataract/	54959
10	exp phacoemulsification/	14524

For peer review only

11	(cataract* or pha?oemulsif*).mp. [mp=title, abstract, heading word, drug trade name, original title, device manufacturer, drug manufacturer, device trade name, keyword, floating subheading word, candidate term word]	91182
12	9 or 10 or 11	92259
13	exp eye surgery/ or exp surgery/	4584292
14	(Surg* or procedure* or operat* or incision*).mp.	6019212
15	13 or 14	7359674
16	(micro or small or minimally or minimal).mp.	2452911
17	(microincision* or micro-incision* or incision*).ti.	18429
18	16 or 17	2465927
19	7 and 12 and 15 and 18	315
20	8 or 19	1138
21	limit 20 to (human and english language and yr="2000 -Current")	724

MEDLINE

Search conducted in May 2020

**Search**	**Query**	**Records Retrieved**

For peer review only

1	exp Glaucoma, Angle-Closure/ or exp Low Tension Glaucoma/ or exp Glaucoma/ or exp Glaucoma, Open-Angle/ or exp Glaucoma, Neovascular/ or exp Glaucoma Drainage Implants/	52351
2	glaucoma*.mp.	70177
3	1 or 2	70398
4	exp General Surgery/	38657
5	(Surg* or procedure* or operat* or incision* or invasive* or penetrat* or Drainage implant surg* or Electrocautery or Cyclophotocoagulation*).mp.	4681137
6	4 or 5	4681137
7	exp Endothelial Cells/	58726
8	endothelial* cell*.ab. or endothelial* cell*.ti.	164910
9	7 or 8	178945
10	3 and 6 and 9	509
11	exp Cataract/	28781
12	exp Phacoemulsification/	9762
13	(cataract* or pha?oemulsif*).mp.	72591
14	11 or 12 or 13	72661

For peer review only

15	exp General Surgery/	38657
16	(Surg* or procedure* or operat* or incision*).mp.	4326472
17	15 or 16	4326472
18	(micro or small or minimally or minimal).mp.	1918877
19	(incision* or (microincision* or micro-incision*)).ti.	15328
20	18 or 19	1930443
21	9 and 14 and 17 and 20	218
22	10 or 21	708
23	limit 22 to (english language and humans and yr="2000 -Current")	408

For peer review only

Appendix II: Draft data extraction form

Reviewer name:	
**Citation details:**<br>Authors:<br>Title:<br>Journal:<br>Year:	
**Study details:**<br>Study design:<br>Sample size:<br>Country:	
**Participant characteristics:**<br>Age:<br>Sex:	

**Inclusion criteria**<br>**Exclusion criteria**	
**Surgery type:**<br>**Intervention:**<br>**Comparator(s):**	
**Outcome**: (at all time assessment points)<br>Mean ECD pre-operation:<br>Mean ECD post-operation:<br>Percentage ECD loss:<br>Corneal complications/adverse events:	

For peer review only

PRISMA-P 2015 Checklist

This checklist has been adapted for use with protocol submissions to *Systematic Reviews* from Table 3 in Moher D et al: Preferred reporting items for systematic review and meta-analysis protocols (PRISMA-P) 2015 statement. *Systematic Reviews* 2015 **4**:1

Section/topic	#	Checklist item	Information reported		Line number(s)
			Yes	No	
**ADMINISTRATIVE INFORMATION**					
**Title**					
Identification	1a	Identify the report as a protocol of a systematic review	☒	☐	5-14
Update	1b	If the protocol is for an update of a previous systematic review, identify as such	☐	☐	NA
**Registration**	2	If registered, provide the name of the registry (e.g., PROSPERO) and registration number in the Abstract	☒	☐	82
**Authors**					
Contact	3a	Provide name, institutional affiliation, and e-mail address of all protocol authors; provide physical mailing address of corresponding author	☒	☐	39-43
Contributions	3b	Describe contributions of protocol authors and identify the guarantor of the review	☒	☐	24-27
**Amendments**	4	If the protocol represents an amendment of a previously completed or published protocol, identify as such and list changes; otherwise, state plan for documenting important protocol amendments	☐	☐	NA
**Support**					
Sources	5a	Indicate sources of financial or other support for the review	☒	☐	34-39
Sponsor	5b	Provide name for the review funder and/or sponsor	☒	☐	34-39
Role of sponsor/funder	5c	Describe roles of funder(s), sponsor(s), and/or institution(s), if any, in developing the protocol	☐	☐	NA
**INTRODUCTION**					
**Rationale**	6	Describe the rationale for the review in the context of what is already known	☒	☐	87-144
**Objectives**	7	Provide an explicit statement of the question(s) the review will address with reference to participants, interventions, comparators, and outcomes (PICO)	☒	☐	141-144
**METHODS**					

For peer review only

BioMed Central
The Open Access Publisher

Section/topic	#	Checklist item	Information reported Yes	No	Line number(s)
**Eligibility criteria**	8	Specify the study characteristics (e.g., PICO, study design, setting, time frame) and report characteristics (e.g., years considered, language, publication status) to be used as criteria for eligibility for the review	☒	☐	145-182
**Information sources**	9	Describe all intended information sources (e.g., electronic databases, contact with study authors, trial registers, or other grey literature sources) with planned dates of coverage	☒	☐	183-194
**Search strategy**	10	Present draft of search strategy to be used for at least one electronic database, including planned limits, such that it could be repeated	☒	☐	183-194
STUDY RECORDS					
Data management	11a	Describe the mechanism(s) that will be used to manage records and data throughout the review	☒	☐	195-262
Selection process	11b	State the process that will be used for selecting studies (e.g., two independent reviewers) through each phase of the review (i.e., screening, eligibility, and inclusion in meta-analysis)	☒	☐	195-262
Data collection process	11c	Describe planned method of extracting data from reports (e.g., piloting forms, done independently, in duplicate), any processes for obtaining and confirming data from investigators	☒	☐	195-262
**Data items**	12	List and define all variables for which data will be sought (e.g., PICO items, funding sources), any pre-planned data assumptions and simplifications	☒	☐	217-229
**Outcomes and prioritization**	13	List and define all outcomes for which data will be sought, including prioritization of main and additional outcomes, with rationale	☒	☐	164-174
**Risk of bias in individual studies**	14	Describe anticipated methods for assessing risk of bias of individual studies, including whether this will be done at the outcome or study level, or both; state how this information will be used in data synthesis	☒	☐	203 -216
DATA					
**Synthesis**	15a	Describe criteria under which study data will be quantitatively synthesized	☒	☐	230-240
	15b	If data are appropriate for quantitative synthesis, describe planned summary measures, methods of handling data, and methods of combining data from studies, including any planned exploration of consistency (e.g., I^2, Kendall's tau)	☒	☐	230-240
	15c	Describe any proposed additional analyses (e.g., sensitivity or subgroup analyses, meta-regression)	☒	☐	230-240
	15d	If quantitative synthesis is not appropriate, describe the type of summary planned	☒	☐	230-240
**Meta-bias(es)**	16	Specify any planned assessment of meta-bias(es) (e.g., publication bias across studies, selective reporting within studies)	☒	☐	246-252
**Confidence in cumulative evidence**	17	Describe how the strength of the body of evidence will be assessed (e.g., GRADE)	☒	☐	246-262

For peer review only

BioMed Central
The Open Access Publisher
